# Estimation of Glomerular Filtration Rate Based on Serum Cystatin C versus Creatinine in a Uruguayan Population

**DOI:** 10.1155/2014/837106

**Published:** 2014-08-24

**Authors:** Inés Lujambio, Mariana Sottolano, Leonella Luzardo, Sebastián Robaina, Nadia Krul, Lutgarde Thijs, Florencia Carusso, Alicia da Rosa, Ana Carina Ríos, Alicia Olascoaga, Mariela Garau, Liliana Gadola, Oscar Noboa, Jan A. Staessen, José Boggia

**Affiliations:** ^1^Unidad de Hipertensión Arterial, Hospital de Clínicas Dr. Manuel Quintela, Universidad de la República, Avenida Italia 2870, 11600 Montevideo, Uruguay; ^2^Departamento de Fisiopatología, Universidad de la República, Montevideo, Uruguay; ^3^Centro de Nefrología, Universidad de la República, Montevideo, Uruguay; ^4^Departamento Laboratorio de Patología Clínica at Hospital de Clínicas, Universidad de la República, Montevideo, Uruguay; ^5^Department of Cardiovascular Sciences, Studies Coordinating Centre, Research Unit Hypertension and Cardiovascular Epidemiology, University of Leuven (KU Leuven), Leuven, Belgium; ^6^Departamento de Métodos Cuantitativos, Facultad de Medicina, Universidad de la República, Montevideo, Uruguay; ^7^Department of Epidemiology, Maastricht University, Maastricht, The Netherlands

## Abstract

*Background*. Estimation of glomerular filtration rate (eGFR) from biomarkers has evolved and multiple equations are available to estimate renal function at bedside. *Methods*. In a random sample of 119 Uruguayans (54.5% women; 56.2 years (mean)), we used Bland and Altman's method and Cohen's kappa statistic to assess concordance on a continuous or categorical (eGFR < 60 *versus* ≥60 mL/min/1.73 m^2^) scale between eGFR_cys_ (reference) and eGFR derived from serum creatinine according to the Modification of Diet in Renal Disease (eGFR_mdrd_) or the Chronic Kidney Disease Epidemiology Collaboration equations (eGFR_epi_) or from both serum cystatin C and creatinine (eGFR_mix_). *Results*. In all participants, eGFR_mdrd_, eGFR_epi_, and eGFR_mix_ were, respectively, 9.7, 11.5, and 5.6 mL/min/1.73 m^2^ higher (*P* < 0.0001) than eGFR_cys_. The prevalence of eGFR <60 mL/min/1.73 m^2^ was the highest for eGFR_cys_ (21.8%), intermediate for eGFR_mix_ (11.8%), and the lowest for eGFR_mdrd_ (5.9%) and eGFR_epi_ (3.4%). Using eGFR_cys_ as reference, we found only fair agreement with the equations based on creatinine (Cohen's kappa statistic 0.15 to 0.23). *Conclusion*. Using different equations we reached clinically significant differences in the estimation of renal function. eGFR_cys_ provides lower estimates, resulting in higher prevalence of eGFR <60 mL/min/1.73 m^2^.

## 1. Introduction

The glomerular filtration rate (GFR) is the most widely used indicator of overall renal function. The GFR can be measured by clearance of an ideal, usually exogenous, filtration marker such as inulin, iothalamate, EDTA, diethylene triamine pentaacetic acid, or iohexol. The clearance of endogenous markers such as creatinine or blood urea nitrogen can overestimate or underestimate the GFR. All these methods have the drawback to be complex and require 24-hour urine collection, which is not always practicable in day-to-day clinical practice. A more pragmatic approach is to estimate GFR from equations based on serum creatinine [1, 2].

More recently, experts proposed cystatin C as an alternative to creatinine [3]. Cystatin C is a nonglycosylated protein with low molecular mass (13.3 kDa) generated by all nucleated cells of the body at a constant rate, is freely filtered by the glomerulus, and is not secreted by renal tubules but completely reabsorbed with subsequent degradation by proximal tubular cells [4, 5]. For those properties cystatin C is an endogenous surrogate of GFR. Compared with serum creatinine, cystatin C levels are less dependent on ethnicity, sex, age, and muscle mass or protein intake. Moreover, as serum creatinine, cystatin C is an independent predictor of cardiovascular and overall mortality [6].

Compared to GFR measurement based on the renal clearance of exogenous markers, estimated glomerular filtration rate (eGFR) is more precise if derived from both cystatin C and creatinine levels in serum [3]. This observation was consistent across sex, age, and diabetes mellitus [7]. On the other hand, measurement of cystatin C is costly and eGFR based on cystatin C needs further validation across a broad spectrum of populations with or without chronic kidney disease. The purpose of our current study was to compare estimates of eGFR based on cystatin C and creatinine in randomly recruited Uruguayans, using eGFR derived from cystatin C as reference.

## 2. Materials and Methods

### 2.1. Study Population

GEnotipo Fenotipo y Ambiente de la HiperTension en UruguaY (GEFA-HT-UY) is a prospective cohort study started in April 2012 conducted by the Unidad de Hipertensión Arterial, Hospital de Clínicas Dr. Manuel Quintela, Universidad de la República, Montevideo, Uruguay [8]. The Ethics Committee of the University Hospital approved the study protocol and all participants gave informed written consent. The aim of the study is to explore the relation of blood pressure with genetic and environmental factors in a representative Uruguayan population sample. Nuclear families were randomly recruited from the inhabitants of a geographically defined area, the Juana de América housing project, located about 10 km from downtown Montevideo. A nuclear family had to include at least one parent and two siblings. The minimum age was 18, without upper age limit. Family members living at the same address or within a distance of no more than 10 km were eligible. We invited participants by telephone. The participation rate among eligible subjects was 72.7%. In November 2013, 149 people had participated, of whom we excluded 30 from the present analysis, because either cystatin C or creatinine had not been measured (*n* = 22) or because they had not yet completed the physical examination (*n* = 8). Thus, the number of participants analysed statistically totalled 119.

### 2.2. Field Work

The examinations took place at a field centre located within the neighbourhood. Trained observers administered a standardised questionnaire inquiring into each participant's medical history, smoking and drinking habits, and intake of medications. They measured blood pressure according to the European guidelines. After participants had rested for 5 minutes in the sitting position, the observers obtained five consecutive blood pressure readings (phase V diastolic pressure) to the nearest 2 mm Hg, using mercury sphygmomanometers. Standard cuffs had a 12 × 24 cm inflatable portion, but if upper arm girth exceeded 31 cm, larger cuffs with 15 × 35 cm bladders were used. Pulse pressure is the difference of systolic minus diastolic blood pressure. Mean arterial pressure is diastolic pressure plus one-third of pulse pressure. For analysis, the five blood pressure readings were averaged. Hypertension was an office blood pressure of at least 140 mm Hg systolic or 90 mm Hg diastolic or use of antihypertensive drugs. The observers measured body height to the nearest 0.5 cm with a pliable measurer and the participant standing against the wall. Participants wore light indoor clothing without shoes for body weight measurements. Body mass index was weight in kilograms divided by square of height in meters. Venous blood samples were obtained after at least 12 hours fasting and were kept at 4°C and within 2 hours period were analysed for serum levels of cystatin C, creatinine, cholesterol, and glucose. Diabetes mellitus was the use of anti-diabetic drugs or a fasting glucose ≥ 126 mg/dL (7 mmol/L).

### 2.3. Arterial Phenotypes

After the participants had rested 15 min in the supine position, we recorded during an 8 s period the radial waveforms at the right side by applanation tonometry. We used a high-fidelity SPC-301 micromanometer (Millar Instruments, Houston, TX) interfaced with a computer running SphygmoCor software, version 8.2 (AtCor Medical, West Ryde, New South Wales, Australia). We discarded recordings when the systolic or diastolic variability of consecutive waveforms exceeded 5% or the amplitude of the pulse wave signal was less than 80 mV. We calibrated the radial pulse wave on the brachial blood pressure [9]. From the radial signal, the SphygmoCor software calculates the aortic pulse wave by means of a validated generalised transfer function [10, 11]. The augmentation index was the ratio of the second to the first peak of the pressure wave expressed as a percentage.

Aortic pulse wave velocity was measured by sequential ECG-gated recordings of the arterial pressure waveform at the carotid and femoral arteries [12]. We measured the distances from the suprasternal notch to the carotid sampling site (distance *A*) and from the suprasternal notch to the femoral sampling site (distance *B*). Pulse wave travel distance was calculated as distance *B* minus distance *A*. Pulse transit time was the average of 10 consecutive beats. Pulse wave velocity was the distance in meters divided by the transit time in seconds [12].

### 2.4. Laboratory Methods

Serum cystatin C was measured by a particle-enhanced turbidimetric immunoassay (PETIA), (COBAS, Roche diagnostics, Germany). The latex enhanced particles coated with anticystatin C antibodies in the reagent agglutinate with the human cystatin C in the sample. The degree of the turbidity caused by the aggregate was determined turbidimetrically at 546 nm. This assay has a detection limit of 0.4 mg/L and a coefficient of variation of 1.3%. Serum creatinine was measured by modified kinetic Jaffé methods with the modifications described elsewhere [13, 14]. The detection limit is 0.17 mg/dL and the coefficient of variation was 1.6%. We use a creatinine method that has calibration traceable to an IDMS reference measurement procedure according to present recommendations [15, 16].

### 2.5. Estimated Glomerular Filtration Rate

We estimated GFR using four equations. First, we computed eGFR from serum cystatin C eGFR_cys_, as described by Inker and colleagues [3]. Next, we calculated eGFR from serum creatinine according to the IDMS-traceable MDRD Study Equation (MDRD) [17, 18] formula (eGFR_mdrd_) or the Chronic Kidney Disease Epidemiology Collaboration (CKD-EPI) [2] equation (eGFR_*epi*⁡_). Finally, as proposed by Inker and colleagues, [3] we also derived eGFR from both serum cystatin C and serum creatinine (eGFR_mix_). All aforementioned estimates [1–3] account for sex and age and with the exception of eGFR_cys_ also consider ethnicity (black* versus* nonblack). This particular characteristic was irrelevant for our current study as our participants only included Whites mainly of European descent. Table S1 in the online data supplement provides detailed information on each formula (see Table S1 in Supplementary Material available online at http://dx.doi.org/10.1155/2014/837106). In our current analyses, we compared findings based on the various methods to estimate GFR against eGFR_cys_ as the referent method. Low glomerular filtration rate (L-GFR) was an eGFR < 60 mL/min/1.73 m^2^ based on a single determination of each biomarker.

### 2.6. Statistical Analysis

For database management and statistical analysis, we used SAS software, version 9.3 (SAS Institute, Cary, NC). First, in exploratory analyses, we assessed the characteristics of participants by fourths of the distribution of eGFR_cys_. For comparison of means and proportions, we applied Student's* t*-test (or ANOVA) and the *χ* ^2^ statistic, respectively. We assessed agreement between paired measurements on a continuous scale by Bland and Altman's method [19]. To allow comparison with literature data, we also computed correlation coefficients. The National Kidney Foundation KDOQI guideline proposes a threshold of 60 mL/min/1.73 m^2^ to diagnose chronic kidney disease [20]. In categorical analyses, we, therefore, also assessed the agreement between equations to dichotomize subjects in L-GFR or not-L-GFR using Cohen's kappa statistic [21]. A kappa value of 0.20 or less indicates slight agreement, 0.20 to 0.40 fair agreement, 0.41 to 0.60 moderate agreement, 0.61 to 0.80 substantial agreement, and 0.81 to 1.00 almost perfect agreement. We studied the association between the four definitions of eGFR dichotomized at 60 mL/min/1.73 m^2^ in hypertensive and diabetic subjects by McNemar's test for paired comparisons of proportions. Because of the low frequencies in some cells, we applied exact statistics in two-by-two tables. Finally, we assessed the added capacity of eGFR_cys_ to differentiate between normotension* versus* hypertension or between people with or without diabetes mellitus, using the integrated discrimination improvement (IDI) and the net reclassification improvement (NRI) [22, 23]. Statistical significance was an *α* level of 0.05.

## 3. Results 

### 3.1. Characteristics of Participants

The 119 participants included 68 women (57.1%) and 53 (44.5%) hypertensive patients, of whom 35 (66.0%) were on antihypertensive drug treatment. Among 68 women and 51 men, 11 (16.2%) and 5 (9.8%) were smokers; 21 women (30.1%) and 32 men (62.7%) reported intake of alcohol. In smokers, median tobacco use was 10 cigarettes per day (interquartile range, 6–15). In drinkers, the median alcohol consumption was 8 g per day (interquartile range, 4–54). In the whole study population, age (SD) averaged was 56.5 (17.3) years and systolic and diastolic blood pressure 126.5 (19.6) mm Hg and 79.6 (11.7) mm Hg, respectively. Based on a self-report of the main maternal and paternal background, 37 participants (31.1%) reported a mixture of Caucasian, African, or Native-American, while 82 participants (68.9%) reported coincident Caucasian background.

Among all participants, serum cystatin C and serum creatinine averaged from 0.99 (0.22) mg/L to 0.81 (0.21) mg/dL with no difference between women and men for cystatin C (0.99* versus* 1.00 mg/L; *P* = 0.77), whereas women had lower serum creatinine than men had (0.72* versus* 0.93 mg/dL; *P* < 0.0001). In all participants, mean values were 80.0 (23.8) mL/min/1.73 m^2^ for eGFR_cys_, 89.7 (22.5) mL/min/1.73 m^2^ for eGFR_mdrd_, 91.5 (19.0) mL/min/1.73 m^2^ for eGFR_epi_, and 85.6 (20.2) mL/min/1.73 m^2^ for eGFR_mix_, with no sex differences (*P* ≥ 0.23).

Table 1 provides the characteristics of participants by fourths of the distribution of eGFR_cys_, which was used as reference. The prevalence of hypertension (*P* = 0.027), age (*P* < 0.0001), and systolic blood pressure (*P* = 0.0097), but not diastolic blood pressure (*P* = 0.79) or mean arterial pressure (*P* = 0.18) increased (*P* = 0.027) with lower eGFR_cys_ category. The central systolic augmentation index and aortic pulse wave velocity also rose (*P* < 0.0001) across decreasing fourths of the eGFR_cys_ distribution. Trends in eGFR_mdrd_, eGFR_epi_, and eGFR_mix_ ran in parallel with the distribution of eGFR_cys_.

### 3.2. Concordance between Estimates of GFR on a Continuous Scale

**Figure 1:**
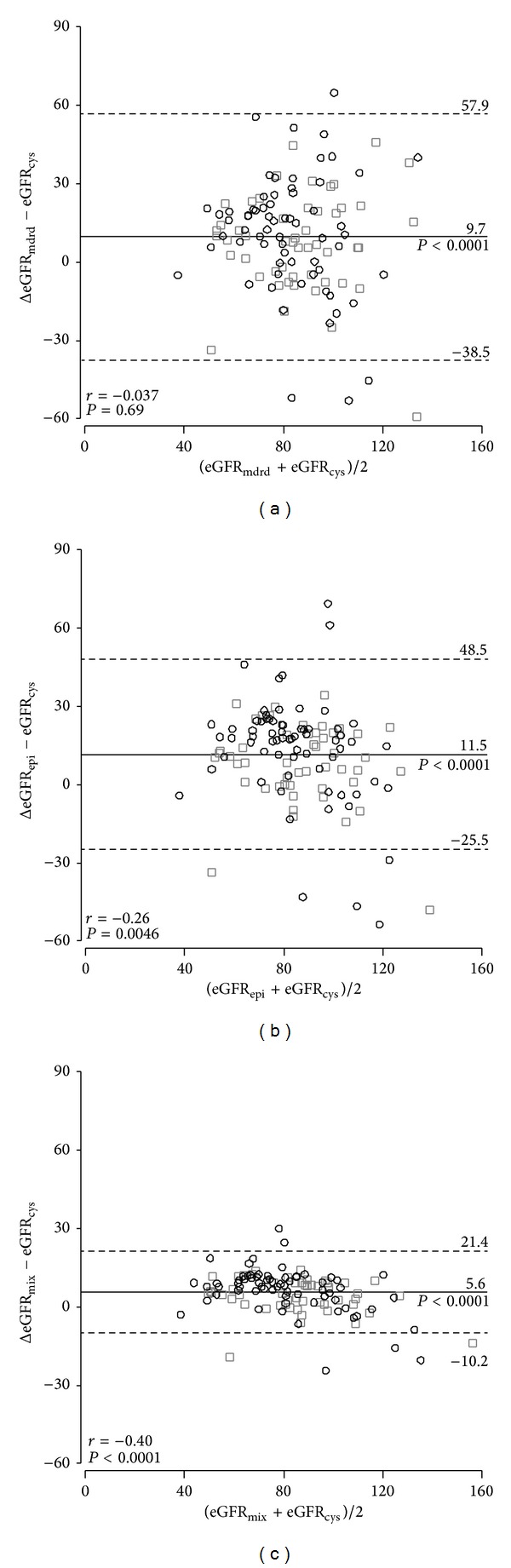
Bland and Altman plots comparing eGFR_mdrd_, eGFR_epi_, and eGFR_mix_, with eGFR_cys_ as the referent method. eGFR_cys_, eGFR_mdrd_, eGFR_epi_, and eGFR_mix_ indicate estimated glomerular filtration rate derived from serum cystatin C, from serum creatinine according to the Modification of Diet in Renal Disease or the Chronic Kidney Disease Epidemiology Collaboration equations, or from both serum cystatin C and creatinine. *R* indicate the intraclass correlation coefficient. *P* denotes the significance level.

**Figure 2:**
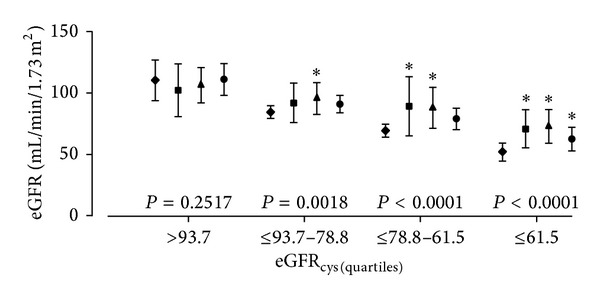
Mean and mean ±1 standard deviation interval of eGFR_cys_ (◆), eGFR_mdrd_ (■), eGFR_epi_ (▲), and eGFR_mix_ (●) across fourths of the distribution of eGFR_cys_. The abbreviations eGFR_cys_, eGFR_mdrd_, eGFR_epi_, and eGFR_mix_ indicate estimated glomerular filtration rate derived from serum cystatin C, from serum creatinine according to the Modification of Diet in Renal Disease or the Chronic Kidney Disease Epidemiology Collaboration equations, or from both serum cystatin C and creatinine. eGFR_mdrd_, eGFR_epi_, and eGFR_mix_ were consistently higher (*P* < 0.0001) than eGFR_cys_ except in the highest category of eGFR_cys_ (*P* = 0.25).

Figure 1 shows the Bland and Altman plots comparing eGFR_mdrd_, eGFR_epi_, and eGFR_mix_, with eGFR_cys_ as the referent method.  Table 2 shows the mean deviations of eGFR_mdrd_, eGFR_epi_, and eGFR_mix_ from eGFR_cys_. In all participants, eGFR_mdrd_, eGFR_epi_, and eGFR_mix_ were, respectively, 9.7, 11.5, and 5.6 mL/min/1.73 m^2^ higher than eGFR_cys_. The corresponding ±2 SD intervals expressed in mL/min/1.73 m^2^ (Figure 1) ranged from −38.5 to +57.9 for eGFR_mdrd_, −25.5 to +48.5 for eGFR_epi_, and −10.2 to +21.4 for eGFR_mix_, and the corresponding correlation coefficients were −0.04 (*P* = 0.69), −0.26 (*P* = 0.0046), and −0.40 (*P* < 0.0001), respectively. Analyses stratified according to sex, age, normotension* versus* hypertension, and absence* versus* presence of diabetes mellitus were consistent with those in all participants (Table 2).  Figure 2 shows that across fourths of the distribution of eGFR_cys_, eGFR_mdrd_, eGFR_epi_, and eGFR_mix_ were consistently higher (*P* < 0.002) than eGFR_cys_ except in the highest category of eGFR_cys_ (*P* = 0.25).

### 3.3. Concordance between Estimates of GFR on a Categorical Scale

The prevalence of L-GFR was the highest for eGFR_cys_, intermediate for eGFR_mix_, and the lowest for eGFR_mdrd_ and eGFR_epi_ (Table 3). Using eGFR_cys_ as reference, Cohen's kappa statistic was 0.230 (95% confidence interval [CI], 0.036 to 0.427; *P* = 0.0005) for eGFR_mdrd_, 0.151 (CI, −0.021 to 0.322; *P* = 0.032) for eGFR_epi_, and 0.587 (CI, 0.399 to 0.775; *P* < 0.0001) for eGFR_mix_.

### 3.4. Association between Chronic Kidney Disease and Response Variables

Among 53 hypertensive patients, the prevalence of L-GFR was higher (*P* < 0.0001) if patients were categorized based on eGFR_cys_ (17 patients, 32.1%) compared with eGFR_mdrd_ (4 patients, 7.5%), eGFR_epi_ (3 patients, 5.7%), or eGFR_mix_ (8 patients, 15.1%). Among 20 diabetic patients, we observed a similar trend. The prevalence of L-GFR was 8 patients (40.0%) based on eGFR_cys_, 2 patients (10.0%) based on eGFR_mdrd_, 2 patients (10.0%) based on eGFR_epi_, and 3 patients (15%) based on eGFR_mix_. However, the differences with eGFR_cys_ did not reach formal statistical significance (*P* ≥ 0.075).

Finally, we explored whether an eGFR_cys_ below 60 mL/min/1.73 m^2^ improved the differentiation between normotension* versus* hypertension or between people without or with diabetes mellitus based on the other estimates of GFR. However, the classification based on eGFR_cys_ did not improve IDI (*P* ≥ 0.53) or NRI (*P* ≥ 0.24) for hypertension or IDI (*P* ≥ 0.37) or NRI (*P* ≥ 0.24) for diabetes mellitus.

## 4. Discussion 

In our current analysis, we compared the performance of the equations based on cystatin C and creatinine to estimate GFR in a Uruguayan population sample. The Uruguayan population has been considered as mainly European descent, with a negligible Native American or African contributions. However, based on serological and molecular markers, recent studies demonstrate that Native American and African had an important influence in the conformation of the present one [24–26].

The key finding was that eGFR_cys_ provides lower estimates in comparison with creatinine based equations (eGFR_mdrd_ and eGFR_epi_). Mean eGFR was 80.0, 89.7, 91.5, and 85.6 mL/min/1.73 m^2^ for eGFR_cys_, eGFR_mdrd_, eGFR_epi_, and eGFR_mix_, respectively. Thus, the prevalence of L-GFR, was higher when derived from equations involving cystatin C (eGFR_cys_ and eGFR_mix_) than when derived from creatinine based equations. The prevalence of L-GFR using eGFR_cys_, eGFR_mdrd_, eGFR_epi_, and eGFR_mix_ was 21.8%, 5.9%, 3.4%, and 11.8%, respectively. In categorical analysis, the agreement between cystatin-based (eGFR_cys_) equations and creatinine-based equations (eGFR_mdrd_ and eGFR_epi_) to detect eGFR under 60 mL/min/1.73 m^2^ was low.

Over the past, many reports highlighted the ability of cystatin C to detect renal disease early in different settings [27–31]. Several researchers reported cystatin C outperforms serum creatinine in the early diagnosis of acute kidney injury [32, 33] or its prognosis [34]. In line with our results, other investigators reported lower estimates of eGFR using cystatin C as biomarker [6, 35, 36]. In contrast, in a Belgian population sample [*n* = 4,189], Delanaye and colleagues report that the prevalence of eGFR below 60 mL/min/1.73 m^2^ using eGFR_cys_, eGFR_mdrd_, eGFR_epi_, and eGFR_mix_ was 4.7%, 13.0%, 9.8%, and 5.0%, respectively [37]. In a recent report, among 394 old (>74 y) subjects and patients from England, Kilbride and colleagues describe lower eGFR_cys_ (55.2 mL/min/1.73 m^2^) in comparison with creatinine based equations (eGFR_mdrd_, 57.6 mL/min/1.73 m^2^; eGFR_epi_, 57.0 mL/min/1.73 m^2^) [38]. Grams and colleagues [36], analyzing representative subsamples of the adult participants in the National Health and Nutrition Examination Surveys (NHANES; 1988–1994 [*n* = 15,133] and 1999–2002 [*n* = 8,238]), observed slightly higher eGFR using cystatin C than creatinine equations (102.9* versus* 99.4 mL/min/1.73 m^2^). However, in line with our results, the proportion of subjects with eGFR below 60 mL/min/1.73 m^2^ was larger if derived from cystatin C than creatinine (5.5%* versus* 4.7%). More recently, Tsai and colleagues [35] reported similar results in diabetic patients enrolled in NHANES. The mean eGFR_cys_ was 100.7 mL/min/1.73 m^2^ and the eGFR_epi_ was 95.1 mL/min/1.73 m^2^ while the proportions of patients with eGFR below 60 mL/min/1.73 m^2^ were 22.0% and 16.5%, respectively. Pattaro and colleagues also found similar results in a cross-sectional analysis of a population from three alpine villages [39]. Finally, in a recent meta-analysis Shlipak and colleagues noticed similar mean eGFR_cys_ and eGFR_epi_ estimates while the proportion of subjects under the threshold to define CKD was 13.7 and 9.7 mL/min/1.73 m^2^ using cystatin and creatinine based equations, respectively [6].

In line with our current results, Delanaye described low agreement between creatinine and cystatin C based formulas (kappa statistic 0.32 [eGFR_cys_ *versus* eGFR_mdrd_] and 0.39 [eGFR_cys_ *versus* eGFR_epi_]) [37]. In this Belgian study, the percentage of discordant subjects estimated by eGFR_mdrd_, eGFR_epi_, and eGFR_mix_ was 11.2%, 8.29%, and 2.71%, taking eGFR_cys_ as reference.

How to explain the discrepancies between cystatin C and creatinine based equations? We first discarded all potential sources of preanalytical and analytical errors. We analysed creatinine in fresh serum samples and cystatin C in samples that were kept frozen at −80°C. Over a period of 10 years, a decay in cystatin C levels occurs using a particle-enhanced nephelometric assay (PENIA) [40]. Such decay is not observed if one uses the more robust particle-enhanced turbidimetric assay (PETIA). Furthermore, our samples were analysed within one year after blood collection and were processed with calibration each time. We used a validated method [40, 41] and reagents (Tina-quant Cystatin C Gen. 2) standardized to the international reference material ERM-DA 471/IFCC, as currently recommended for the use of CKD-EPI equations [3, 7]. Studies of bias (mean difference from reference method) usually overestimated GFR compared with the reference when using MDRD (range −1.0 to +3.5) or CKD-EPI (range −0.23 to +4.4) and underestimated when using cystatin C based equations (range −5.7 to −1.2) [38, 42–44]. Fewer studies showed a lower positive bias of cystatin C than CKD-EPI based equations [7, 45].

Several epidemiological studies showed that cystatin C is a better predictor of outcomes in coronary heart disease, acute coronary syndrome, and heart failure, independently of serum creatinine and GFR estimation [6, 46–49]. Furthermore, Peralta et al. demonstrated in a large and ethnically diverse population that subjects with decreased eGFR_cys_ had elevated risk of death, cardiovascular disease, and heart failure and had an elevated risk of kidney failure [50, 51]. In keeping with these studies, our population sample had a high cardiovascular risk profile. We found a higher prevalence of L-GFR among hypertensive subjects (*P* < 0.001) if they were categorized based on eGFR_cys_ (32.1%) than on eGFR_*epi*⁡_ (5.7%) with a similar but not significant trend among the few diabetics patients. However, when computing IDI and NRI we did not observe significant differences between the various estimates of eGFR for hypertension or diabetes (*P* ≥ 0.24). In accordance with previous reports [48, 52–54], the prevalence of hypertension (*P* = 0.27) and age (*P* < 0.0001), systolic blood pressure (*P* < 0.01), cholesterol (*P* = 0.004), aortic pulse wave velocity (*P* < 0.0001), and the central augmentation index (*P* < 0.0001) increased with lower categories of eGFR_cys_ (Table 1).

Our results should be interpreted within the limitation of the study. First, we did not have a reliable “*gold standard*” due to the variability in 24 h urine collections. Performing inulin or iothalamate clearance implies invasive and tedious procedures that are not suitable for our population study. Second, participants of the study may not be representative of the Uruguayan population, because we randomly sampled a neighbourhood and our participants had a higher cardiovascular risk profile than the general population of Uruguay [55]. Finally, the small sample size of our population is a limiting factor to analyse specific subgroups of participants. However, the number of participants is large enough to describe the difference between eGFR estimating equations.

In conclusion, to our knowledge, this is the first report based on a population from South America comparing to the performance of eGFR equations based on cystatin C and creatinine. We confirm discrepancies in eGFR using equation based on different biomarkers, particularly in the range of GFR under 60 mL/min/1.73 m^2^. Generally, the equation based on cystatin C, compared with creatinine, results in lower eGFR values and, therefore, higher estimates of the prevalence of eGFR below 60 mL/min/1.73 m^2^.

## Supplementary Material

Table S1 describes the equations used to compute the estimated glomerular filtration rate using cystatin C, creatinine or both. Table S2 shows the differences and Table S3 the absolute bias between eGFRmdrd, eGFRepi, or eGFRmix and the reference method (eGFRcys). Table S4 summarize the computations of the eGFR according to racial parental background of the participants.

## Figures and Tables

**Table 1:** Characteristics of 119 participants by fourths of the distribution of eGFR_cys_.

Characteristic	Categories of eGFR_cys_				*P*
eGFR_cys_ limits, mL/min/1.73 m^2^	>93.7	93.7–78.7	78.7–61.5	≤61.5	*⋯*
Number of subjects (%)	30	30	30	29	*⋯*
Women	18 (60.0)	22 (73.3)	12 (40.0)	16 (53.3)	0.063
Smokers	4 (13.3)	5 (16.7)	3 (10.0)	4 (13.8)	0.90
Drinking alcohol	16 (53.3)	13 (43.3)	12 (40.0)	12 (41.4)	0.73
Hypertension	8 (26.7)	11 (36.7)	16 (53.3)	18 (62.1)	0.027
Antihypertensive treatment	3 (10.0)	8 (26.7)	12 (40.0)	12 (66.6)	0.32
Cardiovascular disease	2 (6.7)	1 (3.3)	3 (10.0)	2 (6.9)	0.87
Diabetes mellitus	4 (13.3)	1 (3.3)	7 (23.3)∗	8 (27.6)	0.058
Mean (SD) of characteristic					
Age, years	41.3 (14.0)	52.1 (14.7)^†^	64.2 (13.9)^†^	68.9 (11.8)	<0.0001
Body mass index, kg/m^2^	27.6 (4.4)	29.2 (5.1)	29.1 (5.3)	29.5 (4.6)	0.44
Office blood pressure					
Systolic pressure, mmHg	118.5 (15.1)	123.3 (16.6)	130.9 (21.1)	133.6 (22.0)	0.0097
Diastolic pressure, mmHg	79.3 (10.6)	78.2 (11.9)	79.8 (12.1)	81.4 (12.7)	0.79
Pulse rate, beats per minute	71.3 (7.1)	69.1 (9.9)	75.4 (8.9)∗	71.0 (9.8)	0.054
Central augmentation index, %	14.7 (14.8)	19.6 (11.3)	28.7 (9.8)^†^	23.6 (10.9)	<0.0001
Aortic pulse wave velocity, m/s	7.6 (1.8)	8.6 (2.5)	11.1 (3.2)^†^	10.0 (2.9)	<0.0001
Biochemical measurements					
Total cholesterol (mg/dL)	204.2 (30.2)	194.6 (28.8)	216.5 (38.6)∗	225.3 (34.9)	0.004
Glucose (mg/dL)	100.0 (35.3)	96.6 (12.7)	101.8 (21.6)	101.9 (15.6)	0.88
Serum cystatin C (mg/L)	0.75 (0.15)	0.94 (0.05)^§^	1.03 (0.07)^§^	1.27 (0.14)^§^	<0.0001
Serum creatinine, mg/dL	0.75 (0.14)	0.81 (0.15)	0.77 (0.28)	0.92 (0.23)∗	0.0077
eGFR_mdrd_, mL/min/1.73 m^2^	103.2 (21.6)	92.9 (16.1)∗	90.1 (24.5)	71.9 (5.4)^†^	<0.0001
eGFR_epi_, mL/min/1.73 m^2^	107.1 (14.2)	96.4 (12.8)^†^	88.6 (17.1)∗	73.4 (14.1)^‡^	<0.0001
eGFR_mix_, mL/min/1.73 m^2^	110.4 (13.1)	90.7 (7.0)^§^	78.6 (8.6)^§^	61.8 (9.2)^§^	<0.0001

eGFR_cys_, eGFR_mdrd_, eGFR_epi_, and eGFR_mix_ indicate estimated glomerular filtration rate derived from serum cystatin C, from serum creatinine according to the IDMS-traceable Modification of Diet in Renal Disease (MDRD) Study Equation or the Chronic Kidney Disease Epidemiology Collaboration equations, or from both serum cystatin C and creatinine. Office blood pressure was the average of five consecutive readings. Hypertension was an office blood pressure of ≥140 mmHg systolic or ≥90 mmHg diastolic or use of antihypertensive drugs. Diabetes mellitus was a fasting glucose level of ≥126 mg/dL or use of antidiabetic drugs. The central augmentation index was standardised to a heart rate of 75 beats/minute. Conversion factors: creatinine from mg/dL to *μ*mol/L, multiply by 88.4; cholesterol from mg/dL to mmol/L, multiply by 0.0259. *P* values denote the significance of the differences in prevalence or means across quartiles of eGFR_cys_. Significance of the difference with the adjacent lower fourth:∗*P* ≤ 0.05; ^†^ *P* ≤ 0.01; ^‡^ *P* ≤ 0.001; ^§^ *P* ≤ 0.0001.

**Table 2:** Differences between various estimates of GFR with eGFR derived from serum cystatin C as referent method.

Group	*N*	Difference in estimates of glomerular filtration rate (mL/min/1.73 m^2^)		
		eGFR_mdrd_	eGFR_epi_	eGFR_mix_
All participants	119	9.7 (5.3–14.0)^§^	11.5 (8.2–14.9)^§^	5.6 (4.1–7.0)^§^
Women	68	11.3 (4.7–17.9)^‡^	13.7 (8.7–18.6)^§^	6.4 (4.3–8.5)^§^
Men	51	7.5 (2.1–12.9)^§^	8.7 (4.3–12.9)^‡^	4.5 (2.6–6.3)^§^
<60 years	57	3.2 (−4.1–10.4)	9.6 (3.9–15.3)^†^	4.5 (2.1–6.8)^‡^
≥60 years	62	15.6 (10.8–20.5)^§^	13.3 (9.4–17.2)^§^	6.6 (4.9–8.3)^§^
Normotension	66	8.1 (0.9–15.3)∗	10.4 (4.9–15.8)^‡^	4.9 (2.6–7.3)^§^
Hypertension	53	11.6 (7.3–15.9)^§^	12.9 (9.5–16.4)^§^	6.3 (4.8–7.7)^§^
No diabetes	99	9.5 (4.7–14.4)^‡^	11.9 (8.3–15.7)^§^	5.7 (4.1–7.3)^§^
Diabetes	20	10.3 (−0.7–21.3)	9.4 (0.7–18.1)∗	4.9 (1.4–8.4)^†^

eGFR_mdrd_, eGFR_epi_, and eGFR_mix_ indicate estimated glomerular filtration rate from serum creatinine according to the Modification of Diet in Renal Disease or the Chronic Kidney Disease Epidemiology Collaboration equations or from both serum cystatin C and creatinine. Differences were computed as eGFR_mdrd_, eGFR_epi_, or eGFR_mix_ minus GFR estimated from serum cystatin C (eGFR_cys_). The values between brackets were the 95% confidence intervals (mean ± 1.96 standard errors). *N* indicates the number of participants. Significance of the difference with eGFR_cys_: ∗*P* ≤ 0.05; ^†^ *P* ≤ 0.01; ^‡^ *P* ≤ 0.001; ^§^ *P* ≤ 0.0001.

**Table 3:** Prevalence by categories of eGFR according to the stages of chronic kidney disease, for equation to estimate the GFR.

Stage of kidney disease	Estimated glomerular filtration rate				*P*
	eGFR_cys_	eGFR_mdrd_	eGFR_epi_	eGFR_mix_	
<60 mL/min/1.73 m^2^	26 (21.8)	7 (5.9)^‡^	4 (3.4)^§^	14 (11.8)∗	<0.0001
60–89 mL/min/1.73 m^2^	56 (47.1)	59 (49.6)	51 (42.8)	58 (48.7)	0.73
≥90 mL/min/1.73 m^2^	37 (31.1)	53 (44.5)∗	64 (53.8)^‡^	47 (39.5)	0.004

eGFR_cys_, eGFR_mdrd_, eGFR_epi_, and eGFR_mix_ indicate estimated glomerular filtration rate derived from serum cystatin C, from serum creatinine according to the Modification of Diet in Renal Disease or the Chronic Kidney Disease Epidemiology Collaboration equations or from both serum cystatin C and creatinine. Values are number of participants (%). *P* values are for the overall difference across four estimates of glomerular filtration rate. Significance of the difference with eGFR_cys_:∗*P* ≤ 0.05; ^†^ *P* ≤ 0.01; ^‡^ *P* ≤ 0.001; ^§^ *P* ≤ 0.0001.
