# Supplementary material for: Estimation of Glomerular Filtration Rate Based on Serum Cystatin C versus Creatinine in a Uruguayan Population
Source: Int J Nephrol. 2014 Aug 24;2014:837106. doi: 10.1155/2014/837106 (PMC4158300; doi:10.1155/2014/837106)
Supplement: Supplementary file 1 — Table S1 describes the equations used to compute the estimated glomerular filtration rate using cystatin C, creatinine or both. Table S2 shows the differences and Table S3 the absolute bias between eGFRmdrd, eGFRepi, or eGFRmix and the reference method (eGFRcys). Table S4 summarize the computations of the eGFR according to racial parental background of the participants. [file 837106.f1.pdf]

Supplementary Material

This appendix formed part of the original submission and has been peer reviewed.
We posted it as supplied by the authors.

Supplement to: Lujambio I, *et al*
Estimation of glomerular filtration rate based on serum cystatin C versus creatinine in a Uruguayan population.
International Journal of Nephrology 2014.

Table S1. Estimated glomerular filtration rate derived from serum cystatin C and/or creatinine

Estimated glomerular filtration rate	Cystatin C (mg/L)	Creatinine (mg/dL)	Equation
**$eGFR_{cys}$**			
Women	≤0.8		$133 \times (Scys / 0.8)^{-0.499} \times 0.996^{Age} x\ 0.932$
	>0.8		$133 \times (Scys / 0.8)^{-1.328} \times 0.996^{Age} x\ 0.932$
Men	≤0.8		$133 \times (Scys / 0.8)^{-0.499} \times 0.996^{Age}$
	>0.8		$133 \times (Scys / 0.8)^{-1.328} \times 0.996^{Age}$
**$eGFR_{mdrd}$**			
Women			$175 \times (Scr)^{-1.154} \times Age^{-0.203} \times 0.742$ [× 1.212 if black]
Men			$175 \times (Scr)^{-1.154} \times Age^{-0.203}$ [× 1.212 if black]
**$eGFR_{epi}$**			
Women		≤0.7	$144 \times (Scr/0.7)^{-0.329} \times 0.993^{Age}$ [× 1.159 if black]
		>0.7	$144 \times (Scr/0.7)^{-1.209} \times 0.993^{Age}$ [× 1.159 if black]
Men		≤0.9	$141 \times (Scr/0.9)^{-0.411} \times 0.993^{Age}$ [× 1.159 if black]
		>0.9	$141 \times (Scr/0.9)^{-1.209} \times 0.993^{Age}$ [× 1.159 if black]
**$eGFR_{mix}$**			
Women	≤0.8	≤0.7	$130 \times (Scr/0.7)^{-0.248} \times (Scys/0.8)^{-0.375} \times 0.995^{Age}$ [× 1.08 if black]
		>0.7	$130 \times (Scr/0.7)^{-0.601} \times (Scys/0.8)^{-0.375} \times 0.995^{Age}$ [× 1.08 if black]
	>0.8	≤0.7	$130 \times (Scr/0.7)^{-0.248} \times (Scys/0.8)^{-0.711} \times 0.995^{Age}$ [× 1.08 if black]
		>0.7	$130 \times (Scr/0.7)^{-0.611} \times (Scys/0.8)^{-0.711} \times 0.995^{Age}$ [× 1.08 if black]
Men	≤0.8	≤0.9	$135 \times (Scr/0.9)^{-0.207} \times (Scys/0.8)^{-0.375} \times 0.995^{Age}$ [× 1.08 if black]
		>0.9	$135 \times (Scr/0.9)^{-0.601} \times (Scys/0.8)^{-0.375} \times 0.995^{Age}$ [× 1.08 if black]
	>0.8	≤0.9	$135 \times (Scr/0.9)^{-0.207} \times (Scys/0.8)^{-0.711} \times 0.995^{Age}$ [× 1.08 if black]
		>0.9	$135 \times (Scr/0.9)^{-0.601} \times (Scys/0.8)^{-0.711} \times 0.995^{Age}$ [× 1.08 if black]

Abbreviations: $eGFR_{cys}$, $eGFR_{mdrd}$, $eGFR_{epi}$, $eGFR_{mix}$ indicate estimated glomerular filtration rate derived from serum cystatin C, from serum creatinine according to the Modification of Diet in Renal Disease or the Chronic Kidney Disease Epidemiology Collaboration equations, or from bot serum cystatin C and creatinine. Scys and Scr indicate serum cystatin C and serum creatinine, respectively.

Table S2. Differences between various estimates of GFR with eGFR derived from serum cystatin C as referent method

Group	N	Difference in estimates of glomerular filtration rate (mL/min/1.73m^2)		
		$eGFR_{mdrd}$	$eGFR_{epi}$	$eGFR_{mix}$
All participants	119	9.7 (-38.4–57.8)§	11.5 (-25.5–48.5)§	5.6 (-10.2–21.4)§
Women	68	11.3 (-43.1–65.7)‡	13.7 (-27.1–54.5)§	6.4 (-11.2–24.0)§
Men	51	7.5 (-30.9–45.9)§	8.7 (-22.1–39.5)‡	4.5 (-8.7–17.7)§
<60 years	57	3.2 (-51.4–57.8)	9.6 (-33.2–52.4)†	4.5 (-13.5–22.5)‡
≥60 years	62	15.6 (-22.4–53.6)§	13.3 (-17.5–44.1)§	6.6 (-6.8–20.0)§
Normotension	66	8.1 (-50.3–66.5)*	10.4 (-34.0–54.8)‡	4.9 (-14.1–23.9)§
Hypertension	53	11.6 (-19.8–43.0)§	12.9 (-12.1–37.9)§	6.3 (-4.3–16.9)§
No diabetes	99	9.5 (-39.1–58.1)‡	11.9 -25.3–49.1) §	5.7 (-10.3–21.7)§
Diabetes	20	10.3 (-36.7–57.3)	9.4 (-27.6–46.4)*	4.9 (-10.1–19.9)†

Abbreviations: $eGFR_{mdrd}$, $eGFR_{epi}$, $eGFR_{mix}$ indicate estimated glomerular filtration rate from serum creatinine according to the Modification of Diet in Renal Disease or the Chronic Kidney Disease Epidemiology Collaboration equations, or from both serum cystatin C and creatinine. Differences were computed as $eGFR_{mdrd}$, $eGFR_{epi}$, or $eGFR_{mix}$ minus GFR estimated from serum cystatin C ($eGFR_{cys}$). Values between brackets are the mean ± 2 SD intervals. N indicates the number of participants. Significance of the difference with $eGFR_{cys}$: * P≤0.05; † P≤0.01; ‡ P≤0.001; § P≤0.0001.

Table S3. Absolute bias between various estimates of GFR and the eGFR derived from serum cystatin C as referent method.

Group	N	Absolute bias in estimates of glomerular filtration rate (mL/min/1.73m^2)		
		$eGFR_{mdrd}$	$eGFR_{epi}$	$eGFR_{mix}$
All participants	119	15.6 (2.9–53.0)	17.0 (1.1–46.0)	7.8 (0.9–18.6)
Women	68	17.4 (2.9–55.4)	18.5 (2.6–46.7)	8.9 (1.3–20.5)
Men	51	10.9 (2.8–44.9)	12.2 (0.4–33.4)	5.7 (0.7–14.0)
<60 years	57	10.9 (0.4–64.7)	16.2 (1.0–53.7)	6.6 (1.0–24.5)
≥60 years	62	17.4 (3.8–44.9)	17.8 (1.3–33.4)	8.4 (0.9–15.8)
Normotension	66	19.4 (2.9–58.9)	18.1 (1.2–48.0)	8.3 (1.0–20.5)
Hypertension	53	12.3 (1.6–40.4)	15.6 (1.1–28.4)	6.7 (0.9–12.1)
No diabetes	99	15.7 (1.6–55.4)	17.4 (1.2–46.0)	8.1 (1.0–19.1)
Diabetes	20	14.1 (3.5–52.2)	14.6 (0.7–44.3)	7.2 (0.5–17.2)

Values are median and the 5th to 95th interval between brackets. N indicates the number of participants. Abbreviations: $eGFR_{mdrd}$, $eGFR_{epi}$, $eGFR_{mix}$ indicate estimated glomerular filtration rate from serum creatinine according to the Modification of Diet in Renal Disease or the Chronic Kidney Disease Epidemiology Collaboration equations, or from both serum cystatin C and creatinine. Absolute bias were computed as the absolute difference between $eGFR_{mdrd}$, $eGFR_{epi}$, or $eGFR_{mix}$ minus GFR estimated from serum cystatin C ($eGFR_{cys}$).

Table S4. Estimated Glomerular Filtration Rate by Racial Parental Background of the Participants.

Group	N	glomerular filtration rate (mL/min/1.73m^2)			
		$eGFR_{cys}$	$eGFR_{mdrd}$	$eGFR_{epi}$	$eGFR_{mix}$
All participants	119	80.0±23.8	89.7±22.5	91.5±19.0	85.6±20.2
Coincident white	82	78.1±25.7	89.6±23.5	90.7±19.7	84.1±21.9
White-Black	17	88.3±14.3	99.7±23.4	100.2±14.5	94.6±14.2
White-Native American	16	77.3±14.4	81.4±18.1	86.9±21.0	81.3±15.6
Black-Native American	4	82.1±7.1	88.8±5.4	95.4±13.7	88.9±9.3

Values are mean ± SD. Abbreviations: $eGFR_{mdrd}$, $eGFR_{epi}$, $eGFR_{cys}$, $eGFR_{mix}$ indicate estimated glomerular filtration rate from serum creatinine according to the Modification of Diet in Renal Disease or the Chronic Kidney Disease Epidemiology Collaboration equations, or from both serum cystatin C and creatinine.
